# A192 CONSUMPTION OF FOOD LOWER IN TRYPTOPHAN IS ASSOCIATED WITH SIGNIFICANT ANXIETY IN PATIENTS WITH CELIAC DISEASE

**DOI:** 10.1093/jcag/gwab049.191

**Published:** 2022-02-21

**Authors:** V Gupta, A Jivraj, D Armstrong, E Verdu, M I Pinto-Sanchez

**Affiliations:** Farncombe Family Digestive Health Research Institute, McMaster University Faculty of Health Sciences, Hamilton, ON, Canada

## Abstract

**Background:**

Tryptophan, an essential amino acid found in many protein-based foods, has been involved in the pathogenesis of mood disorders and celiac disease (CeD). However, dietary tryptophan consumption has not been investigated in CeD.

**Aims:**

To estimate 1) differences in tryptophan content in food consumed in CeD patients compared to matched healthy controls (HC), and 2) whether consumption of tryptophan is associated with the presence of anxiety and depression in patients with CeD.

**Methods:**

This prospective observational study examined adult patients with a diagnosis of CeD, based on positive specific serology and confirmed by duodenal biopsies, enrolled in the Celiac Registry at McMaster University. A group of sex matched HC were recruited from within the community. Participants completed several questionnaires to evaluate, gastrointestinal symptoms (GSRS), Anxiety and Depression (HADS), and dietary intake of tryptophan (Modified Food Frequency Questionnaire). In addition, CeD patients were assessed for celiac disease activity (CSI), gluten-free diet (GFD) adherence (CDAT) and nutritional status. HADs >11 denoted significant anxiety or depression. Cut-off for low vs high amount of tryptophan content in food was established based on the median tryptophan food content in the HC population.

**Results:**

A total of 443 participants were enrolled in the study; 222 with CeD and 221 controls. The majority of participants were female (74.3%) and the adherence to the GFD was very good (Median CDAT=13; IQR 11–15). The CeD patients were older than HC (Median age, yrs. CD= 40 vs HC= 35; p<0.01). Patients with CeD consumed higher amount of tryptophan compared with HC (Median tryptophan g/day CeD=4.41 vs HC=3.31; p<0.01). There were 157 active CeD patients (CSI ≧ 30). There was no difference in tryptophan consumption between patients with and without active CeD (OR=1.021; 95%CI 0.86–1.21; p=0.88). There was no association between tryptophan consumption and gastrointestinal symptoms or depression scores. Fifteen % of the overall population and 18% of the CeD population had anxiety. There was an increased risk of anxiety in the overall population that consumed low amount of tryptophan (OR=1.87; 95%CI 1.01–3.11; p=0.04), and the risk was greater in the CeD population (OR=4.57; 95%CI 1.26–16.41; p<0.01).

**Conclusions:**

Consumption of food with lower amounts of tryptophan in patients with CeD is associated with significant anxiety, which may influence disease management. Future studies should evaluate whether increasing tryptophan in the diet compared to supplements improves outcomes in CeD population, especially in those with anxiety.

**Funding Agencies:**

None

